# Novel genetic defects in primary ciliary dyskinesia affecting function of dynein arms and central pair apparatus

**DOI:** 10.1186/2046-2530-1-S1-O1

**Published:** 2012-11-16

**Authors:** H Omran

**Affiliations:** 1 Klinik und Poliklinik für Kinder - und Jugendmedizin - Allgemeine Pädiatrie - Universitätsklinikum Münster, Germany

## 

Primary ciliary dyskinesia (PCD) is a genetically heterogeneous recessive disorder characterized by defective cilia and flagella motility. Chronic destructive airway disease is caused by abnormal respiratory tract mucociliary clearance. Abnormal sperm flagella propulsion contributes to male infertility. Genetic defects in most PCD patients cause randomization of left-right body asymmetry; approximately half show situs inversus or situs ambiguous. Almost 70 years after the *hy3* mouse carrying *Hydin* mutations was described as a recessive hydrocephalus model, we report recessive *HYDIN* mutations in PCD patients without hydrocephalus. Interestingly, routine TEM analyses were not diagnostic and only high-speed videomicroscopy could confirm correct diagnosis. In addition we report recessive *CCDC103* mutations in another PCD variant characterized by randomization of left/right body asymmetry and partial ODA defects. High-resolution immunofluorescence microscopy analysis documented absence of distal axonemal ODA complexes.

